# Functional diversity of topological modules in human protein-protein interaction networks

**DOI:** 10.1038/s41598-017-16270-z

**Published:** 2017-11-23

**Authors:** Guangming Liu, Huixin Wang, Hongwei Chu, Jian Yu, Xuezhong Zhou

**Affiliations:** 1 School of Computer and Information Technology and Beijing Key Lab of Traffic Data Analysis and Mining, Beijing Jiaotong University, Beijing, 100044 China; 2 Dalian University of Technology, Dalian, 116024 China; 3 Dalian Institute of Chemical Physics, Chinese Academy of Sciences, Dalian, 116023 China

## Abstract

A large-scale molecular interaction network of protein-protein interactions (PPIs) enables the automatic detection of molecular functional modules through a computational approach. However, the functional modules that are typically detected by topological community detection algorithms may be diverse in functional homogeneity and are empirically considered to be default functional modules. Thus, a significant challenge that has been described but not elucidated is investigating the relationship between topological modules and functional modules. We systematically investigated this issue by initially using seven widely used community detection algorithms to partition the PPI network into communities. Four homogeneity measures were subsequently implemented to evaluate the functional homogeneity of protein community. We determined that a significant portion of topological modules with heterogeneous functionality exists and should be further investigated; moreover, these findings indicated that topologically based functional module detection approaches must be reconsidered. Furthermore, we found that the functional homogeneity of topological modules is positively correlated with their edge densities, degree of association with diseases and general Gene Ontology (GO) terms. Thus, topologically based module detection approaches should be used with caution in the identification of functional modules with high homogeneity

## Introduction

Cellular functions are mostly conducted in a highly modular manner^1^ in the context of a molecular interaction network^2^ whose underlying universal laws may potentially be elucidated by advanced approaches derived from network biology^3^. Investigation of the modular organization of interactome networks, such as protein-protein interactions (PPIs), may facilitate further explorations of the underlying molecular network mechanisms that drive human diseases^4,5^, This network medicine framework provides a global system-level view for discovering the potential causes of human diseases and obtaining a better understanding of the correlation between each disease and its molecular functional communities^6,7^, These interaction networks may be used to predict gene function^8^, new disease-associated genes^9^ and the overlapping relationships among disease phenotypes^10,11^, The tacit assumption of network medicine^12^ is that perturbations of a specific protein functional community in the PPI network will result in a disease phenotype^13^. Therefore, the disease module^6,12^, a particular neighborhood with tightly linked proteins associated with a specific phenotype, may be identified from the PPI network through topological network analysis. Kwang-ll Goh *et al*.^6,11,14^, have discovered that the corresponding protein products of the disease genes are more likely to participate in the same functional module and that proteins associated with the same disorder increase the likelihood of sharing similar biological functions; these findings have been revalidated in several other related works^4^.

To date, most disease module detection algorithms have been built on the basis of the findings of topological modules as functional modules with respect to a specific disease. Ruan *et al*.^15^ have used the famous network partition approach (referred to as the GN algorithm) to a colon cancer microarray dataset and have obtained the functional modules that cause colon cancer. Spirin and Mirny^16^ have applied three methods for group identification in the PPI network and have subsequently shown that these topological clusters correspond to protein complexes and functional modules. A clique percolation approach has been used by Zhang *et al*.^17^ to identify protein communities, and the most of their topological modules correspond to functional modules. A graph entropy approach for the identification of functional modules from the PPI network has been proposed by Kenley *et al*.^18^. These previously described methods have generated functional modules from topological modules; therefore, it is assumed that topological, functional and disease modules overlap. Thus, the functional modules correspond to topological modules^12^. As a result of the increased availability of PPI data and molecular functional information, it would be interesting to revisit this issue and investigate the extent to which the functional homogeneity of genes corresponds to their topological interactions.

The main contribution of this study is to investigate the functionally diverse homogeneity of topological protein modules. We initially selected seven well-investigated community algorithms for detecting topological modules in the PPI network. We determined that most modules had fewer than 10 proteins and that the modules significantly overlapped. Second, we simultaneously conducted a homogeneity analysis for each module with Gene Ontology (GO) and pathways and determined that homogeneity also exhibited a diverse distribution. Finally, we analyzed two causes of functional diversity of the modules: disease-related genes and GO term levels.

## Results

### Topological modules of the human PPI network

We investigated the underlying modular structure in the human protein-protein interaction network derived from STRING9 by adopting seven well-studied community detection algorithms (BGLL, Incremental BGLL (IBGLL), Newman Spectral (NS), Label Propagation (RAK), Walktrap (WT), Link Community (LC) and ClusterONE (CO); see the Materials and Methods section). Different methods yielded different protein communities with different sizes and protein memberships, thus potentially influencing our evaluation results. To validate the consistency of the community detection results produced by the different algorithms, we calculated the overlap of the communities generated by these seven methods.

**Figure 1:**
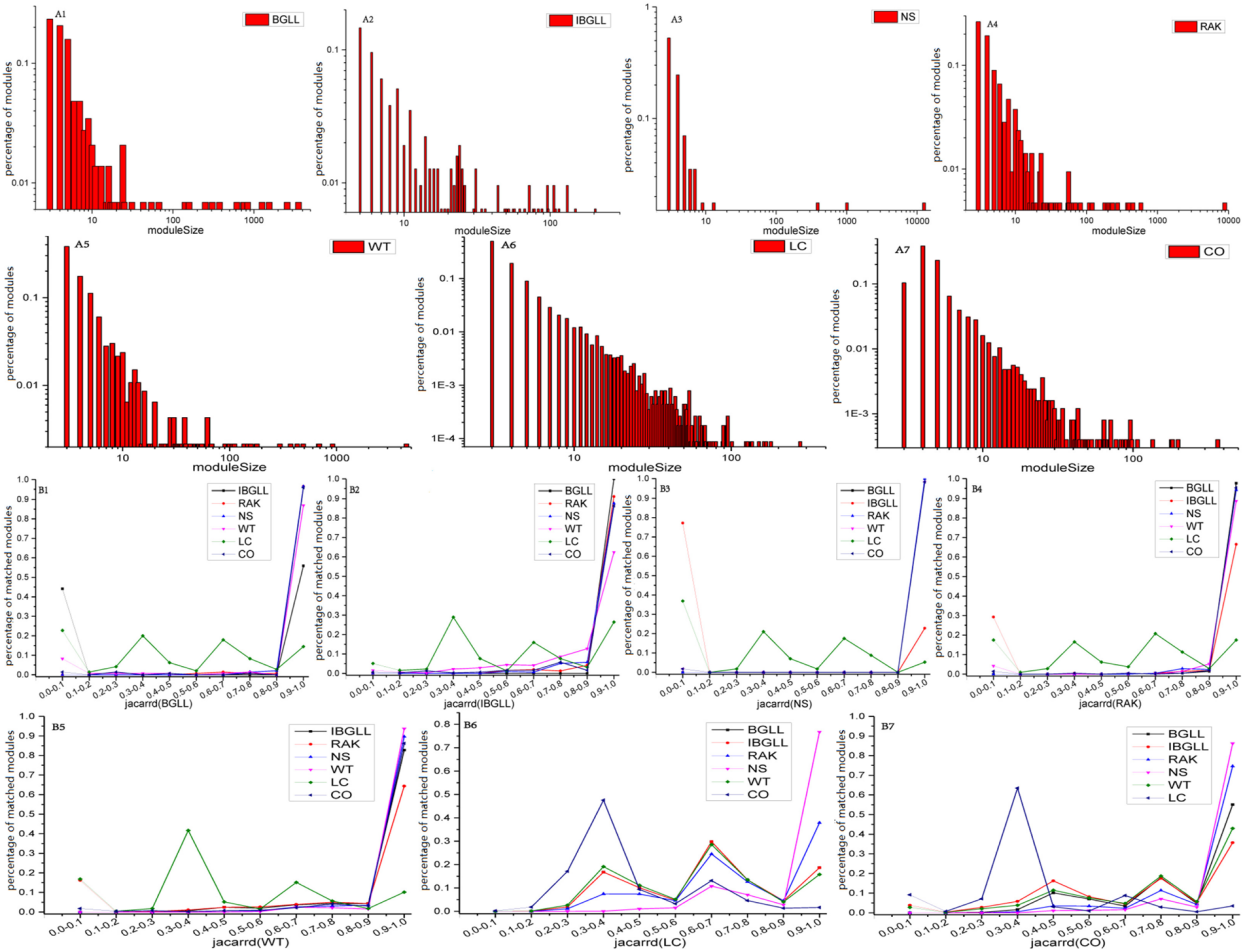
Distribution of module size and the overlap among modules. Figs A1-7 illustrate the distribution of the size of modules detected by seven different community partition methods (BGLL, IBGLL, NS, RAK, WT, LC and CO) in the PPI (String 9) network. The x-axis represents the size of module, and the y-axis describes the percentage of modules. B1–7 indicate the consistency of the community results that detected by all seven community detection methods. The x-axis represents the Jaccard similarity metric between two modules, and the y-axis represents the percentage of matched modules.

As a result, we initially recognized that the proportion of small modules was larger than that of big modules for each method (as indicated in Fig. 1A), thus suggesting that small modules (with size < 10) composed most of the network (41.1%, BGLL; 77.9%, IBGLL; 93%, NS; 73.6%, RAK; 83.4%, WT; 91.1%, LC; 36.5%, CO) in all methods. Moreover, the module size distribution of overlapping module detection methods (LC and CO) approximately followed a power-law distribution, whereas the module size distributions of the other five non-overlapping community detection algorithms had longer tails than the other two distributions. However, the total number of modules produced by each method varied from 57 to 11,387. For example, the NS algorithm generated only 57 modules with a size greater than 2, whereas a protein group exists for 12,527 proteins (14,380 proteins in the String9 database). LC identified 11,387 communities with a size greater than 2, and the protein clusters overlapped. Table 1 presents an account of the communities and the largest module size in all methods.

**Table 1:** The number of modules and the largest module size with respect to seven distinct approaches.

*Methods*	Number of modules	Largest module size
*BGLL*	145	3567
*IBGLL*	314	305
*NS*	57	12527
*RAK*	212	8845
*WT*	463	4510
*LC*	11387	275
*CO*	2486	305

These modules have also been considered to be functional modules in past decades^19^. Second, an underlying modular structure naturally existed in the PPI network, thus indicating that the modules detected by different algorithms shared most of the common protein members. The consistency of the module families among all algorithms was measured through the Jaccard similarity metric, which evaluates significant overlap between paired sets of modules. A high Jaccard value indicates that the module sets of a specific algorithm are highly involved in other module families produced by a distinct algorithm. The results regarding the relationship between the Jaccard similarity intervals and the percentage of protein modules accompanied by different methods are presented in Fig. 1B; these results indicated that community structure/modularity was a fundamental property of the PPI network, as has been described by Zhang^20^ and Rives^21^. These modules generated by LC and CO (Fig. 1B6–B7)were easily contained by other modules that were detected by non-overlapping algorithms. Moreover, the proportion of modules with Jaccard similarity metrics less than 0.1 was quite small for IBGLL (Fig. 1B2), RAK (Fig. 1B4) and WT (Fig. 1B5); however, BGLL (Fig. 1B1) and NS (Fig. 1B3) resulted in a relatively higher proportion than IBGLL at this Jaccard interval, whereas the modules produced by NS and BGLL matched each other well. The reason for this finding is that the modules generated by IBGLL were based on BGLL, and modules with size smaller than 3 were discarded; thus, the absence of proteins contributed to the lower Jaccard metric. According to the above analysis, regardless of whether an overlapping or non-overlapping module detection algorithm was used, the most prominent consequence of these two findings was the presence of various densely linked modules that held the overall PPI network together.

### Evaluating the homogeneity of topological protein modules

#### The reliability of GO and pathway homogeneity

**Figure 2:**
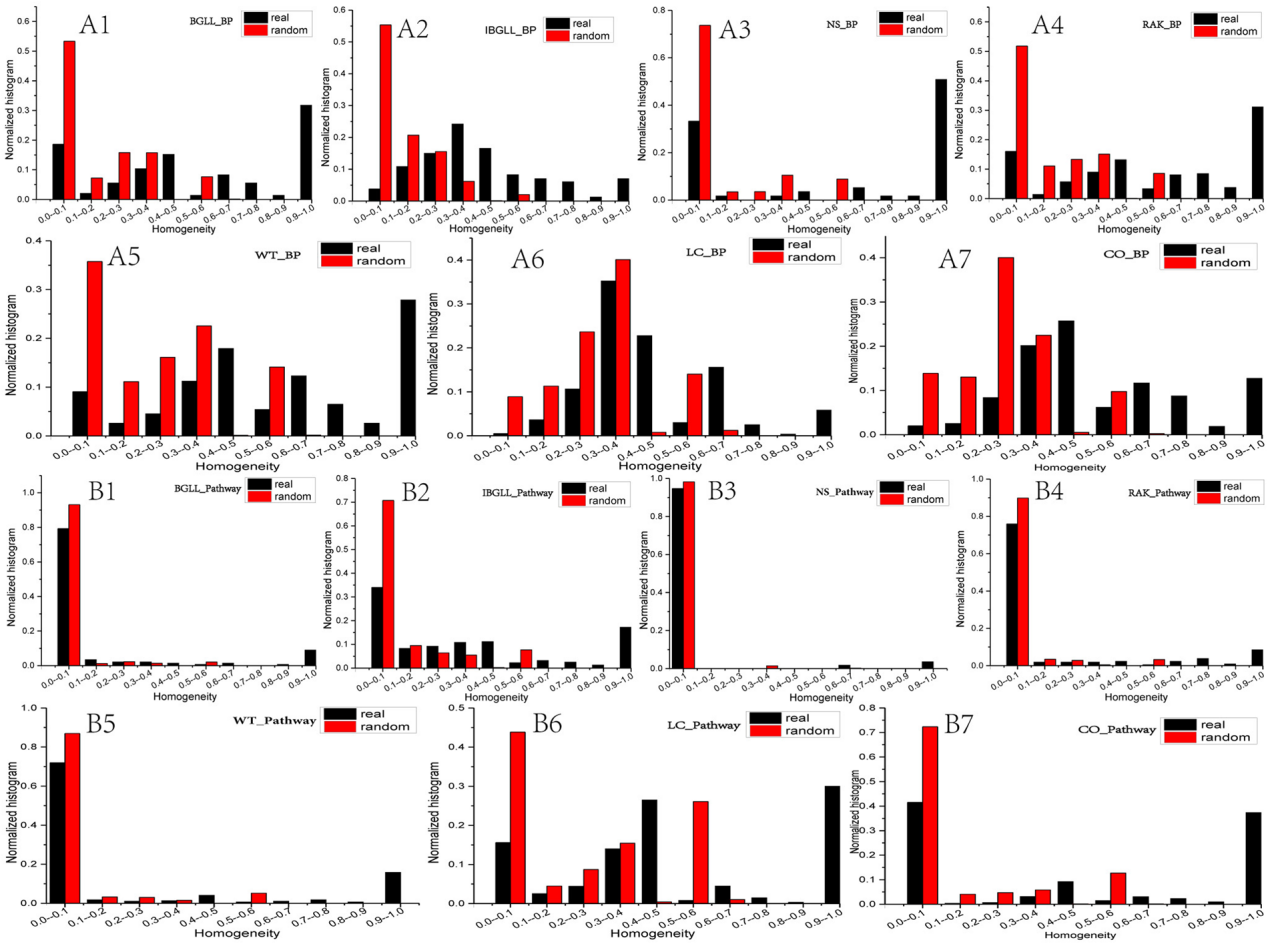
Homogeneity of BP and pathway associations compared with random control. Figs A1–7 illustrate the BP homogeneity comparisons between real and random control for all seven methods. Figs B1–7 show the pathway homogeneity comparisons between real and random control for all seven methods.

Proteins showing dense interaction with one another in one module should have the same or similar functions and be described as having shared commonalities in their biological functional characteristics^22^. We investigated the functional homogeneity of the topological modules in the PPI network by calculating the GO homogeneity and pathway homogeneity for each module by using Equations (3) and (4) (see the Materials and Methods section). A larger value indicates relatively higher homogeneity. Furthermore, to investigate how well the discovered community structures reflected biological functions, the homogeneity results were compared with random expectations (refer to the Materials and Methods section). Finally, we determined that the topological modules exhibited excellent homogeneity compared with the expected modules without advanced planning. Fig. 2A and B depict the comparison of biological process (BP) and pathways, respectively, and the comparison results for cellular component (CC) and molecular function (MF) are shown in Supplementary Fig. 1. For example, consider method IBGLL, in which the value 0.6 (or bigger) can be considered a relative larger homogeneity value. We determined that 21.3% of the modules have a homogeneity larger than 0.6 in BP, as compared with the random control(p = 5.17E-30, chi-square test) (Fig. 2A2). This finding indicated that the proteins in densely connected sub-graphs exhibited a high tendency to share common biological functions^16^. However, we also found that the number of protein modules with lower homogeneity values was greater than the number of modules with higher homogeneity in terms of the GO or pathway associations. For example, only 67 of the 314 modules produced by IBGLL had homogeneity values greater than a relative higher homogeneity 0.6. In summary, the topological modules may have a greater proportion of homogeneous modules than the random controls; however, a substantial proportion (78.7%,IBGLL) of heterogeneous modules also existed. Thus, the distribution of module homogeneity is varied, and the biological functions of the topological modules are diverse.

#### The relationship between the size and density of the modules and homogeneity

**Figure 3:**
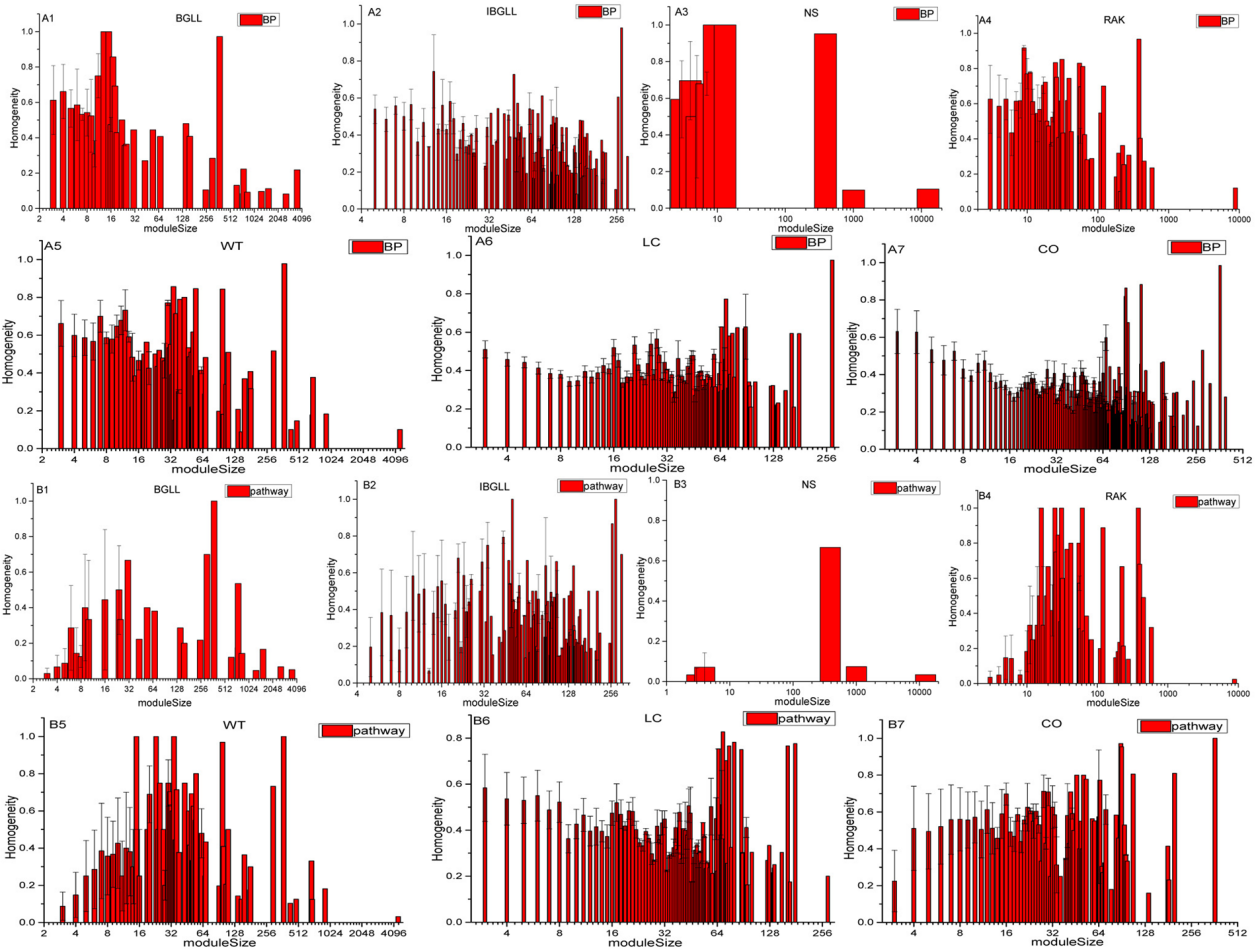
Homogeneity of BP and pathway associations at different module sizes. Figs. A1–7 illustrate the correlation between homogeneity and module size according to GO for all seven methods. Figs B1–7 denote the correlation between homogeneity and module size according to pathway for all seven methods.

Homogeneity varied across the topological modules because small modules (size < 10) represented the largest proportion of all modules; thus, the Pearson correlation coefficient (PCC) and its corresponding p-value (Table 2) were calculated to separately evaluate the underlying correlation between the size and density of the modules and homogeneity with respect to the BP, CC, and MF. As a result, we found that module size was negatively correlated with homogeneity, thus indicating that the topological modules may obtain relatively higher homogeneity if they possess fewer protein members, and vice versa. Given the substantial number of modules generated by each algorithm, the mean and variance of the homogeneity modules of the same size were calculated. Figure 3A presents the distribution of homogeneity related to BP terms, and Supplementary Fig. 2 presents the distribution of homogeneity related to MF and CC terms. A diverse distribution of homogeneity existed in different module sizes. The methods BGLL, NS, RAK and WT detect big modules (with size > 1000) and they have relatively lower PCC between module size and homogeneity in the meantime. In order to quantify how these super modules affect the correlation between module size and homogeneity, we recalculate the PCC and its corresponding p-value by removing super modules (Supplementary Table 1). And we find that the correlation between module size and GO homogeneity have a little change except NS because the biggest module has 12527 proteins in NS and the most modules have less than 10 proteins. That means the methods which detect large modules will give rise to the relatively lower PCC between module size and homogeneity. Furthermore, the same results were obtained according to pathway for the LC and CO methods only; the results obtained from the other five non-overlapping methods indicated that the module size and pathway homogeneity had limited relevance. The reason for the lack of correlation between module size and pathway homogeneity may be that super modules existed in the module sets produced by these non-overlapping algorithms, and we recalculate the PCC and p-value by removing the super modules (size > 1000) and finally we find the module size and pathway homogeneity have positive relationship (Supplementary Table 1). This indicates the relatively larger modules are tend to include more proteins in one pathway and have relative higher homogeneity simultaneously. Furthermore, the number of pathways (1513) was relatively small in the Pathway Interaction Database (PID) database, thus possibly providing another explanation.

**Table 2:** Correlation between module size and homogeneity. PCC is the Pearson Correlation Coefficient between the size and homogeneity and p-value is the significance level.

*Method*	BP	CC	MF	Pathway
*BGLL*	−0.23 (0.01)	−0.15(0.08)	−0.19(0.02)	0.02(0.79)
*IBGLL*	−0.28(2.99E-07)	−0.21(2.36E-04)	−0.32(5.86E-09)	0.02(0.68)
*RAK*	−0.11(0.13)	−0.09(0.18)	−0.09(0.19)	0.00(0.99)
*NS*	−0.15(0.25)	−0.12(0.39)	−0.11(0.39)	0.00(0.97)
*WT*	−0.12(0.01)	−0.09(0.04)	−0.10(0.03)	0.00(0.97)
*LC*	−0.10(9.03E-27)	−0.10(3.86E-26)	−0.16(6.32E-67)	−0.11(1.21E-29)
*CO*	−0.23(2.81E-17)	−0.18(8.29E-11)	−0.26(3.34E-21)	−0.01(0.76)

Proteins exert their functions through interactions with one another^23–25^, the PCC (Table 3) and its corresponding p-value between the edge density and homogeneity were calculated to measure the relationship between edge density and homogeneity. We determined that edge density and homogeneity are positively correlated. Furthermore, we identified an inverse result for pathway homogeneity for nearly all methods (Table 3). This finding indicates that high density modules may tend to participate in diverse pathways. Moreover, community detection methods may fail to detect the disease modules with high pathway homogeneity because a high edge density is one of their main principles pursued. This failure may be caused by the relatively longer average distance between protein pairs in the pathway, which would not have been considered in topological modules. The results of the shortest path lengths in topological modules and pathways confirmed this observation because proteins in pathways tended to have substantially higher average shortest path lengths than topological modules (3.82 vs 2.50, respectively, p-value = 7.43E-118, t-test) according to the IBGLL method. As mentioned before, the big modules (with size > 1000) were detected by BGLL, NS, RAK and WT, we recalculate the PCC between edge density and homogeneity by removing these super modules. Finally, we find that the PCC values have a little decrease for all these four methods (Supplementary Table 2). That means the methods which detect large modules will give rise to the relatively lower PCC between edge density and homogeneity. Overall, we concluded that community detection methods based on topological features may be better suited for identifying functional modules with neighborhood structures (e.g., protein complexes), whereas these methods may not be suitable for the detection of functional modules as pathways.

**Table 3:** Correlation between edge density and homogeneity. PCC is the Pearson Correlation Coefficient between edge density and homogeneity and p-value is the significance level.

*Method*	BP	CC	MF	Pathway
*BGLL*	0.20(0.01)	0.20(0.02)	0.15(0.07)	−0.29(4.44E-04)
*IBGLL*	0.36(3.13E-11)	0.31(1.36E-08)	0.40(1.26E-13)	−0.09(0.13)
*RAK*	0.12(0.08)	0.16(0.02)	0.17(0.01)	−0.15(0.03)
*NS*	−0.01(0.93)	0.13(0.32)	0.17(0.21)	−0.13(0.32)
*WT*	0.19(3.50E-05)	0.20(1.49E-05)	0.12(0.01)	−0.22(1.06E-06)
*LC*	0.21(2.17E-115)	0.03(2.05E-04)	0.02(0.08)	0.03(0.00)
*CO*	0.52(2.29E-91)	0.42(3.01E-56)	0.44(3.14E-59)	−0.19(3.45E-12)

#### Module distance and phenotypic similarity

Phenotypic similarity is another metric used to measure the homogeneity of modules, as discussed by Ghiassian^26^. According to the investigation of disease module hypothesis^10^, the distance between disease modules should be negatively correlated with phenotypic similarity. In recent s, a substantial number of studies have indicated that proteins contribute to diseases with similar phenotypes tend to interact with one another more frequently^27–29^, Therefore, two modules with correspondingly similar phenotypes are assumed to have a relatively shorter topological distance in PPIs. Similarly, when two topological modules are cohesive in their common functional similarity principles, the previously described assumption should be true. Thus, the topological distance between a pair of modules and the phenotypic similarity between them were independently calculated to test this assumption (refer to the Materials and Methods section). However, interestingly, there were mostly positive correlations (e.g., PCC = 0.44, BGLL) between the distances and phenotypic similarities of topological modules (Table 4) with non-overlapping methods but that this correlation became weak with overlapping algorithms. And we find that the methods BGLL, NS, RAK and WT which detect large modules (with size > 1000) have relatively higher PCC, then we recalculate the PCCs by removing super modules. We find that the PCC values have a little change for all these four methods (Supplementary Table 3). That means the methods which detect large modules will give rise to the relatively higher PCCs between distance and phenotype similarity. This finding indicated that the molecular interactions between modules have counterintuitive correlations with their shared phenotypes, thus suggesting that there will be gaps in determining the functional modules directly from topological modules. Furthermore, this disagreement may in turn be a result of the following: (1) the incompleteness of the currently available PPI, the noise interplay between proteins^30^ and the biased protein-protein interactions present in the PPI network^31^ and (2) the potential for the proteins in one module to participate in more than one biological process, thus resulting in widely different phenotypes within one module. The results clearly indicated that the functional diversity distribution of topological modules existed for phenotypes, and further studies are necessary to investigate the complicated relationships between topological modules and functional modules.

**Table 4:** Correlation between module distance and phenotypic similarity. PCC is the Pearson Correlation Coefficient between module distance and phenotypic similarity and p-value is the significance level.

*Method*	PCC(p-value)
*BGLL*	0.44(<1E-127)
*IBGLL*	0.11(1.27E-127)
*RAK*	0.38(<1E-127)
*NS*	0.23(2.48E-20)
*WT*	0.31(<1E-127)
*LC*	5.61E-03(<1E-127)
*CO*	0.12(<1E-127)

### Disease-related modules have higher homogeneity

The detected protein communities provide insights into the methods for identifying the potential biological mechanisms of protein interactions^32^. Our work also revealed the diverse distribution of biological homogeneity within these modules. Furthermore, we determined that the denser edges of a module may contribute to greater homogeneity, whereas many studies have recognized that disease-associated proteins tend to exhibit more dense interactions with one another than with the other proteins in the PPI^33^. Thus, in this study, the proportion of disease-causing proteins located in one specific module was used to validate the potential associations between diseases and module homogeneity. For each module, we searched a disease that occupied the maximum fraction of proteins in one module and then identified the correlation between the ratio and homogeneity. Finally, we discovered that functional homogeneity had a mildly positive correlation with the maximum portion of disease-related genes (PCC = 0.20, p-value = 4.58E-04, BP, IBGLL; Table 5), thus indicating that when more proteins contributed to a common disorder within a topological module, they were typically accompanied by greater functional homogeneity. However, this positive correlation was not significant (p-value >= 0.05) for the BGLL, RAK and NS methods in terms of BP, CC and MF. According to the module size results in a previous work, the non-significant correlation may be caused by super modules (Table 1). The sizes of the largest modules were 3567 (BGLL), 8845 (RAK) and 12,527 (NS), whereas there were 14,380 proteins in the PPI network. The IBGLL method repartitioned the super modules (size >= 400) into multiple, relatively small modules, and significance emerged for all three branches in the GO analysis. Furthermore, we recalculate the PCCs between the percentage of disease-related proteins and homogeneity by removing super modules which are generated in BGLL, RAK, NS and WT (Supplementary Table 4). We find that the values of PCC are decrease that means the methods which detect large modules will give rise to the relatively lower PCC between percentage of disease-related genes and homogeneity. In conclusion, the modules that contain the most proteins related to a specific disease may exhibit greater homogeneity to some extent. This result was consistent with the disease module hypothesis and a recent investigation of disease module detection^26^ which has specified that disease modules are scattered across the entire PPI network rather than being located in only one uniform super module.

**Table 5:** Correlation between percentage of disease-related proteins and homogeneity. PCC is the Pearson Correlation Coefficient between pecentage of disease-related proteins and homogeneity and p-value is the significance level.

*Method*	BP	CC	MF	Pathway
*BGLL*	0.12(0.14)	0.04(0.65)	2.55E-03(0.98)	0.01(0.95)
*IBGLL*	0.20(4.58E-04)	0.10(0.07)	0.20(2.68E-04)	0.19(6.63E-04)
*RAK*	0.13(0.06)	0.14(0.05)	0.12(0.08)	−0.04(0.55)
*NS*	0.14(0.30)	0.04(0.76)	0.01(0.96)	0.02(0.85)
*WT*	0.14(2.74E-03)	0.13(4.12E-03)	0.10(0.03)	−0.05(0.30)
*LC*	0.21(4.07E-118)	0.19(6.71E-95)	0.28(8.49E-204)	0.17(6.70E-79)
*CO*	0.27(9.12E-04)	0.16(2.34E-04)	0.24(2.57E-13)	0.20(3.59E-04)

### GO term generality contributes to higher homogeneity

**Figure 4:**
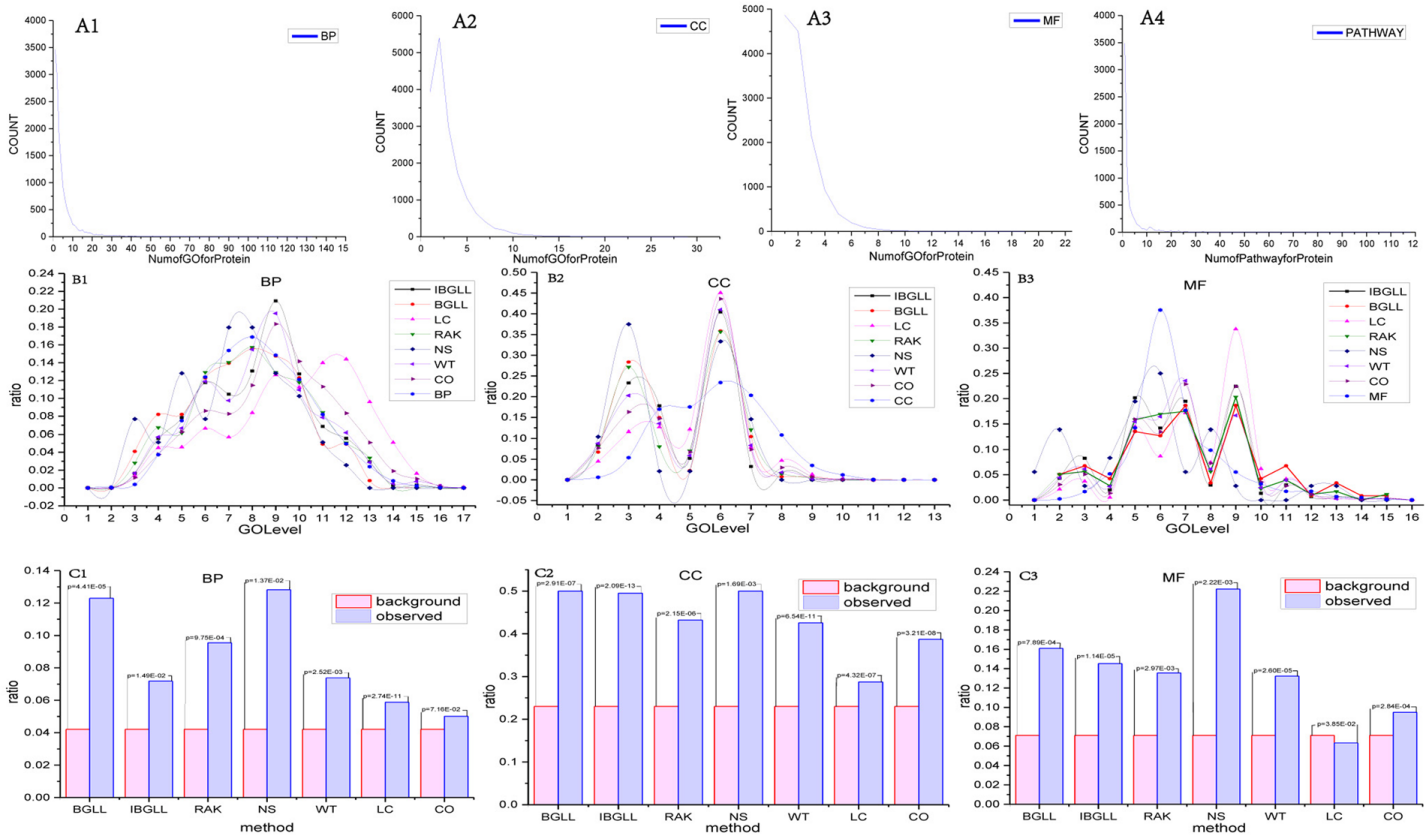
GO and pathway properties and GO level distribution. The underlying reasons for the diverse biological meaning of modules were examined from three aspects. Figures A1–4 indicate the distribution of GO terms and proteins. Figures B1–3 indicate the distribution of GO term levels, thus resulting in higher homogeneity in modules (for each method) in terms of BP, CC and MF. Figures C1–3 indicate the significance of general GO terms by module enrichment, and the pink bars indicate the background ratio of GO terms (level > = 4) with a ratio of the number of modules for each method for the ratio of the number of modules for each method; the p-value denotes the significance of the difference between the two ratios according to a chi-square test.

Each protein within the PPI network is typically annotated by multiple GO terms. We determined that the distribution of the number of GO annotations for genes had a fat-tail distribution (Fig. 4A 1–3), thus indicating that most (44.6%, BP; 55.3%, CC; 71%, MF) proteins were annotated by 1-2 GO terms and that proteins (26.7%, BP; 10.5%, CC; 2.7%, MF) annotated with more than 5 GO terms indeed existed. If each protein in the modules of the PPI network were to have a substantial number of GO annotations, we would expect greater functional homogeneity in these modules. Therefore, we classified the proteins on the basis of their number of GO annotations (e.g., proteins with only 1 GO annotation) and calculated the fraction of proteins of each type in each module. In contrast to common expectations, the fraction of proteins with a low number (particularly one) of GO annotations in a given module had a strong positive correlation with the homogeneity of the module (e.g., BGLL with PCCs: 0.41, 0.31 and 0.30 for BP, CC and MF homogeneity, respectively, Table 6). The only exception is the LC method, which may be a result of its detection of overlapping communities at small scales (90% of modules had less than ten proteins). Considering the fact that super modules detected by BGLL, RAK, NS and WT, we recalculate PCCs by removing super modules and we find that PCCs have a little change for all methods (Supplementary Table 5). We have found that the methods which generate relatively smaller number of modules will give the bigger PCCs (for example: 0.60, NS). This finding may be due to general GO annotations, which are implicitly included in the parental categories of an annotated GO term for genes. We further evaluated the degree to which GO generality might contribute to the homogeneity of topological modules by examining the correlation between the tree-level of GO annotations of proteins in modules and their homogeneity.

**Table 6:** Correlation between the number of GO terms at different levels and homogeneity in terms of BP, CC, MF and pathway. PCC is the Pearson Correlation Coefficient between percentage of proteins annotated by r GO terms and homogeneity and p-value is the significance level.

Number of proteins annotated by (participated in) r GO terms(pathways)		PCC(p-value)			
		BP	CC	MF	pathway
BGLL	1	0.41(3.25E-07)	0.31(1.34E-04)	0.30(2.44E-04)	0.76(2.59E-28)
	2	0.25(2.33E-03)	0.13(1.16E-01)	0.36(1.04E-05)	0.35(1.69E-05)
	3	0.14(1.01E-01)	0.01(9.21E-01)	0.21(1.19E-02)	0.24(3.81E-03)
	4	0.09(2.94E-01)	0.10(2.22E-01)	−0.02(7.88E-01)	0.04(6.57E-01)
	>=5	−0.09(2.80E-01)	−8.24E-04(9.92E-01)	−0.05(5.62E-01)	0.27(9.30E-04)
IBGLL	1	0.25(8.95E-06)	0.15(7.34E-03)	0.09(1.18E-01)	0.58(5.65E-30)
	2	0.05(3.34E-01)	0.12(4.16E-02)	0.10(7.17E-02)	0.27(8.23E-07)
	3	0.15(8.49E-03)	0.05(3.37E-01)	0.04(5.19E-01)	0.16(5.22E-03)
	4	0.04(5.20E-01)	0.03(5.90E-01)	0.05(3.95E-01)	0.07(2.21E-01)
	>=5	−0.21(1.68E-04)	−0.12(3.09E-02)	−0.04(4.57E-01)	0.10(9.22E-02)
RAK	1	0.42(2.07E-10)	0.15(3.02E-02)	0.30(7.73E-06)	0.81(9.07E-50)
	2	0.24(3.62E-04)	0.15(3.27E-02)	0.22(1.31E-03)	0.29(1.96E-05)
	3	0.14(3.88E-02)	0.07(3.20E-01)	0.21(2.07E-03)	0.22(1.30E-03)
	4	0.14(4.43E-02)	0.11(1.05E-01)	0.09(1.82E-01)	0.26(1.50E-04)
	>=5	0.003(9.67E-01)	0.10(1.61E-01)	0.07(3.41E-01)	0.24(3.27E-04)
NS	1	0.60(7.61E-07)	0.37(4.10E-03)	0.55(9.51E-06)	0.82(4.83E-15)
	2	0.30(2.22E-02)	0.27(3.99E-02)	0.39(2.66E-03)	0.01(9.62E-01)
	3	0.18(1.93E-01)	0.04(7.86E-01)	0.25(6.12E-02)	0.01(9.61E-01)
	4	0.09(5.13E-01)	0.17(2.02E-01)	−0.04(7.63E-01)	0.40(1.94E-03)
	>=5	0.03(8.29E-01)	0.05(7.30E-01)	0.01(9.50E-01)	0.41(1.71E-03)
WT	1	0.27(4.90E-09)	0.11(1.70E-02)	0.31(8.91E-12)	0.81(8.46E-107)
	2	0.18(7.31E-05)	0.10(2.98E-02)	0.18(1.32E-04)	0.37(2.44E-16)
	3	0.05(2.93E-01)	0.07(1.15E-01)	0.09(4.84E-02)	0.21(6.72E-06)
	4	0.09(5.68E-02)	0.09(6.30E-02)	0.07(1.40E-01)	0.16(6.86E-04)
	>=5	0.004(9.38E-01)	0.04(3.75E-01)	0.02(7.32E-01)	0.13(3.72E-03)
LC	1	−0.09(3.15E-22)	−0.19(9.22E-91)	−0.06(2.97E-10)	0.31(1.98E-256)
	2	−0.03(4.66E-04)	−0.11(4.94E-30)	0.03(2.33E-04)	0.13(9.62E-43)
	3	−0.04(1.80E-05)	0.02(1.23E-02)	0.05(3.28E-07)	0.07(1.55E-12)
	4	0.04(1.67E-05)	0.06(3.89E-09)	0.07(7.85E-14)	0.08(5.69E-19)
	>=5	0.12(1.37E-39)	0.19(1.41E-90)	0.10(2.28E-24)	0.07(7.26E-14)
CO	1	0.16(1.68E-08)	0.11(4.83E-05)	0.06(2.49E-02)	0.49(1.64E-76)
	2	0.06(2E-02)	0.03(2.2E-02)	0.13(2.69E-06)	0.28(3.71E-24)
	3	0.05(6E-02)	0.02(4.01E-02)	0.10(2.00E-03)	0.17(4.28E-09)
	4	0.01(6.46E-02)	0.04(2.08E-02)	−0.02(4.22E-01)	0.10(2.93E-04)
	>=5	−0.08(3.87E-03)	-0.08(6.47E-03)	−0.04(1.22E-01)	0.14(1.01E-06)

When we considered the tree structure of GO, the percentage of modules at each level exhibited a diverse distribution (Fig. 4B 1–3), and a larger fraction of modules (14.5%, IBGLL) obtained greater homogeneity in terms of high-level GO terms (level is<=4). We further confirmed these findings by classifying the modules and GO terms into two categories according to level 4 and determining the significance (chi-square test) between them (Fig. 4C 1–3). The findings indicated that the general GO terms consistently contributed to greater homogeneity instead of indicating a specific biological meaning.

In addition, we evaluated the statistical magnitude of the proteins by counting proteins that participated in a specific pathway and the distribution among them, which approximately followed a fat-tail distribution (Fig. 4A4). The same result was obtained for the GO terms in this study, thus indicating that the general pathway contributed to greater homogeneity (Table 6).

## Discussion

Most biological functions arise from interactions among many molecular components, which typically form functionally related modules to exert their activities^3,16,34^, The identification of functional modules is a critical process for understanding the potential mechanism of molecular interactions within cells and the underlying mechanisms of complicated disease phenotypes^4,35^, Fortunately, the availability of various types of large-scale interactome networks^36^, such as PPI, signal transduction networks and metabolic networks, have paved the way for the prediction of biological functions using network-based approaches^8,24^.

It has been well established that the relevant genes of similar disease phenotypes have a significantly higher tendency to interact with each other and to have a higher degree of related functions than do random cases^5^. These related studies have developed several network medicine assumptions and/or principles, such as the disease module phenomenon, the consistency between diseases with shared phenotypes and their underlying molecular interactions^12^, and the overlap of topological, functional and disease modules. The overlap assumption indicates that functional modules correspond to topological modules, and a disease may be viewed as the breakdown of a functional module. Most previous studies have indicated that a disease module tends to be a functional and topological module. However, this relationship would not naturally be an inverse one. Thus, molecular interactions exert biological functions and may be used for functional predictions of proteins; however, topological modules detected solely through community discovery methods have a substantial gap that must be filled before they can be considered functional disease modules. In this manuscript, we attempted to address this issue by systematically investigating the functional homogeneity of topological modules extracted by seven widely used community detection methods from a large-scale human PPI network. We determined that the small modules comprised a substantial fraction of all modules, thus indicating a general shortcoming of community detection methods for topological module discovery. Moreover, we determined that the functional properties of topological modules are diverse and heterogeneous; thus, although most topological modules tend to be functionally homogeneous compared with random controls, there are several unavoidable factors, such as edge density, associated disease phenotypes and general GO terms, that contribute to the questionable tendency of functional homogeneity. Furthermore, when we used a recently proposed measure of disease molecular relationships, which has been shown to be a robust measure of disease module overlap, we determined that the molecular distance between topological modules positively correlated with the phenotypic similarity between topological modules. This finding indicated that a greater molecular distance between topological modules is associated with greater phenotypic similarity. Although this result is clearly counterintuitive, it might represent another detectable gap distinguishing topological modules from functional modules.

To the best of our knowledge, this study is the first systematic analysis of the differences between topological modules and their corresponding biological functions and the contributing factors related to the questionably high tendency of functional homogeneities. In this manuscript, we used only two overlapping community detection methods (LC and CO); therefore, the biological functions that may correspond to the overlapping structures should be further investigated. The correlation between distance and phenotypic similarity across modules might change when additional overlapping methods, such as CFinder^37^, Potts model^38^. Lin *et al*.^39^ have found that a topological module usually contains core and ring components and that the major biological function is exerted through core components; thus, it is necessary to consider these core components when detecting functional modules. Furthermore, we also determined that the average shortest path in the modules (i.e., 2.25 in IBGLL) was shorter than that in the pathways (i.e., 3.82 in PID), because topological modules contain only proteins exhibiting dense interaction. Thus, a combination of other valuable biological and topological information may facilitate the effective clustering of non-adjacent proteins^40^ into one module as a new pathway.

## Methods

In this study, we mainly utilized five databases, namely, String9 (Protein-Protein interaction database)^41^, GO^42^, PID (Pathway Interaction Database)^43^, Disease-Connect database^44^ and SemRep^45^. The PPI network was constructed with the String9 database, which indicates the interactions between pairs of proteins. GO and PID were independently used to conduct the enrichment and homogeneity analyses for the topological protein modules. The well-established Disease-Connect disease-related gene dataset was simultaneously used in this study to investigate the relationship between protein topological modules and the diseasome.

### Data Set

#### Protein-Protein Interaction Data

The protein-protein interaction dataset was obtained from the STRING database^46^, and version 9 of the STRING database (String9) was downloaded from the website^41^. This PPI database contains curated known and predicted protein-protein interactions. There is a score value for each protein-protein interaction, and a high score is associated with greater confidence in the protein pair’™s interactions. In our study, we managed the acquisition of high quality interactions within human cells by performing pretreatment of the String9 dataset according to the interactions with scores greater than 700^47^ and the proteins whose identifiers began with the string “9606”. Thus, 14,380 proteins and 218,163 protein-protein interactions were ultimately selected.

#### Gene Ontology

A battery of controlled and structured vocabularies (referred to as ontologies) was used to describe gene products, as provided by Gene Ontology^42^. Moreover, free text definitions and stable unique identifiers were assigned to each term in the GO database. The structure of the Gene Ontology terms was organized as a tree. There were three non-overlapping categories: BP, CC and MF, included in the Gene Ontology; the roots of the three categories were GO:000815 (BP), GO:0005575 (CC) and GO:0003674 (MF), and the corresponding hierarchical heights were 17, 13 and 16, respectively, as described by the GO Consortium. The properties of a specific protein are denoted by these three domains, such that BP describes the biological goals, CC describes the locations and MF describes the activities. There are 40,848 GO terms in the database, including 26,598 biological process slims, 3653 cellular component slims, and 10,697 molecular function slims. Intuitively, the GO terms at a lower level are relatively farther level from the root in the GO hierarchy^48^ and give rise to more specific functional annotations for proteins, whereas the higher-level terms indicate more abstract functional annotations.

#### Pathway database

The pathway database utilized in this study to verify the homogeneity of the topological protein com- munities was PID (Pathway Interaction Database)^43^. PID is composed of three other well-established pathway databases, including NCI-Nature curated data, BioCarta data and Reactome data. There are various molecule types in all three databases; however, only molecules with a corresponding molecule type marked as “protein” or “protein complex” were considered to meet the requirements of our study. Thus, we extracted 1513 pathways from PID, of which 223 pathways were selected from the NCI-Nature curated database, 254 pathways were collected from the BioCarta database, and 838 pathways were obtained from the Reactome^49^ database.

#### Disease-Gene association data

DiseaseConnect (http://disease-connect.org/) is a public web-server for the analysis and visualization of comprehensive knowledge regarding common molecular mechanism-based disease-disease connectivity^44^. The disease-gene relationships from GeneRIF, GeneWays and OMIM were contained in the Disease-Connect database. We ultimately extracted 4551 disease-gene relationships.

#### Disease-Phenotype association data

We extracted the disease-phenotype relationships from SemRep^50^, which identifies semantic predictions from free biomedical text. The semantic predictions extracted from SemRep formed a repository referred to as SemMedDB^45^, which contained approximately 82.2 million predictions. We used the table referred to as Concept to extract the disease name and phenotype name, and the relationships among them were subsequently determined from the table PREDICATION ARGUMENT. Finally, we extracted 6438 items regarding the disease-related phenotype.

### Topological module detection methods

#### Modularity

The community structure, which indicates the phenomenon of densely linked clusters of nodes with sparser edges between them, is a common property of many complex networks^51^. In the past decade, there have been numerous algorithms to detect communities on the basis of the optimization of a metric referred to as modularity, a prominent formulation introduced by Newman and Girvan^52^ that is expressed as follows:

1 $$Q=\frac{1}{L}\times \sum _{i,j\in V}[{M}_{ij}-\frac{{d}_{i}{d}_{j}}{L}]\times {\rm{\Delta }}({C}_{i},{C}_{j})$$

where M is the adjacency matrix that describes the protein interaction network as a graph, $L={\sum }_{i,j\in V}{M}_{ij}$ is the sum of weights of all edges in the graph, V denotes the set of nodes in network, ${d}_{i}={\sum }_{j\in V}{M}_{ij}$ indicates the degree of node i, *C*_*i*_ represents the community that node i belongs to, and Δ function Δ(*u*, *v*) is equal to 1 if u = v and is equal to 0 otherwise. The value of Q was used to measure the strength of modules identified by the community detection algorithms^53^.

#### BGLL

We obtained the topological protein modules by applying the BGLL algorithm, proposed by Vincent D Blondel *et al*.^54^, to protein-protein interaction networks and precisely partitioned the protein-protein interaction network into modules with nodes that were densely inter-connected.

The best partition of the network was accompanied by the highest modularity value; the aim of the BGLL algorithm is to identify the greatest Q by optimizing function (1). There are two phases that are repeated iteratively in the BGLL algorithm. In the beginning, each node was given a different unique community; whether node i was removed into its neighbor’™s community depended on the gain of modularity, which was calculated as follows (2),

2 $${\rm{\Delta }}Q=[\frac{{l}_{in}+2{d}_{i,in}}{L}-{(\frac{{l}_{all}+{d}_{i}}{L})}^{2}]-[\frac{{l}_{i,in}}{L}-{(\frac{{l}_{all}}{L})}^{2}-{(\frac{{d}_{i}}{L})}^{2}]$$

where *l*_*in*_ is the sum of the weights of the edges of the network, *l*_*all*_ is the sum of the weights of the edges incident to the nodes in the network, *d*_*i*_ is the sum of the weights of the edges incident to node i, *d*_*i,in*_ is the sum of the weights of the edges from i to nodes in the network and L is the double of the sum of the weights of all edges in the network. If the Δ*Q* > 0, then the two communities are merged into one community. This first phase stops when no movement of an individual node increases the value of the modularity.

A new network in which the nodes are the communities attained from the first phase are constructed in the second phase. The weights of the edges between nodes in the new network are obtained by summing the weights between the relevant communities in the first phase. The two steps are repeated iteratively until there is no more gain in Q.

#### IBGLL(Incremental BGLL)

Considering the number of genes associated with one disease, modules with more than 400 proteins should be repartitioned. Thus, we propose a novel approach based on BGLL to partition the PPI network into various small modules with sizes under 400 proteins. There are two steps in this algorithm. First, the sub-network from the PPI was extracted with communities with over 400 proteins, on the basis of the modules detected by BGLL. Second, the algorithm referred to as BGLL was iteratively applied to the sub-graphs to obtain smaller communities. The algorithm converged when there was no module size greater than 400.

#### NS(Newman Spectral)

Newman drew his inspiration from graph partition and subsequently proposed a modularity-based optimization community detection algorithm in terms of the spectral attributes of the real network^51^. wo steps are involved in the method. First, the network is split into two sub-graphs in terms of the next-to-largest eigenvalue of the modularity matrix. Second, the modules identified in step 1 are partitioned into two modules according to the additional modularity matrix. These two steps are repeated until there is no positive eigenvalue for the modularity matrix.

#### RAK(Label Propagation)

Raghavan *et al*.^55^ have proposed a localized community detection algorithm referred to as RAK that is mainly for use in understanding information diffusion. Each vertex in the network is initially assigned a unique numeric label. The label for each node is substituted with the label that is dominated by its neighboring nodes. The algorithm converges when all vertex labels do not change. Finally, the vertices that share the same label comprise a community.

#### WT(Walktrap)

Based on the idea of random walk, a module detection algorithm with a hierarchical structure referred to as WT was designed by Pascal Pons *et al*.^56^. A new distance metric of two vertices and communities introduced by a transition matrix is used to capture topological similarities between them. A node is initially considered one community and subsequently merges two adjacent clusters into a new community in terms of the Wards method. The distance between modules is subsequently updated according to the new partition. Thus, the method terminates when only one community is reserved. In this study, the random walk length was t = 4, and the best partition was selected according to the maximal modularity.

#### LC(Link Community)

The previously described methods consider only node grouping, and the detected communities are non-overlapping. However, a protein may have multiple biological functions; thus, the identification of communities with overlap requires substantial work. In contrast to the methods that consider nodes alone, a hierarchical overlap cluster algorithm referred to as link community^57^ is presented. In this method, the similarity between links is initially calculated, and a hierarchical clustering algorithm is subsequently used to build a dendrogram in which each leaf represents an edge from the PPI network. Finally, the tree is cut according to a partition density D (in contrast to the modularity, which endures a resolution limit) to obtain the best level of the most relevant communities.

#### CO(ClusterONE)

Nepusz *et al*.^58^ have proposed an overlapping protein complex detection algorithm that discovers protein complexes more accurately than MCL, MCODE and CFinder. There are three main steps in CO. First, the protein with the highest degree is selected as a seed, and then, a cohesiveness measure is used to determine whether appending or removing proteins can identify densely connected communities of proteins. Second, if the degree of overlap between two communities is higher than a given threshold, then they are merged into a new community. In the third step, modules with fewer than three proteins or modules with a density below a given threshold are abandoned. After these three steps, the overlapping protein complexes are finally detected.

### Functional homogeneity analysis

#### Homogeneity analysis

For each protein topological cluster, we calculate the homogeneity^5^ according to GO and pathway associations. For each module, the maximum fraction of proteins that share the same Gene Ontology annotation (or pathway) was referred to as the GO homogeneity (or pathway homogeneity); According to this definition, the GO homogeneity is calculated by Equation (3):

3 $${H}_{GO}=ma{x}_{i}\,[\frac{{N}_{{G}_{i}}}{{N}_{G}}]$$

where *N*_*G*_ denotes the number of proteins within one protein module annotated by any GO term, and ${N}_{{G}_{i}}$ is the number of proteins within one protein module that shares the ith GO term. The pathway homogeneity was calculated by equation (4):

4 $${H}_{P}=ma{x}_{i}\,[\frac{{N}_{{P}_{i}}}{{N}_{P}}]$$

where *N*_*P*_ is the number of proteins within one protein community that participates in any pathway, and ${N}_{{P}_{i}}$ is the number of proteins within one protein module that participates in the ith pathway.

#### Homogeneity of random control

For a group of proteins, we reassigned the GO (pathway) terms to annotate each protein by chance with the same number of its inherent hold. The process was as follows: if a protein was annotated by m GO terms in the source database, we randomly assigned m GO terms to this protein as its annotated GO terms. In the same way, if a protein participated in n pathways, we randomly designated n pathways to this protein. In this study, we generated 100 random instances to approach statistical significance for all seven distinct community detection algorithms.

#### Molecular distance between topological modules

The distance between two communities was employed to verify the topological similarity between them, and the metric introduced by Jorg^10^ was used to measure the network-based separation of two disease modules. The distance between two modules A and B was calculated by comparing the mean shortest distance <*d*_*AA*_> and <*d*_*BB*_> of proteins within the corresponding topological modules to the mean shortest distance <*d*_*AB*_> between their proteins, as computed by Equation (5).

5 $${s}_{AB}= < {d}_{AB} > -\frac{ < {d}_{AA} > + < {d}_{BB} > }{2}$$

#### Symptom similarity

We investigated the phenotypic similarity between two topological protein modules by constructing the phenotype vectors of each topological module and calculating the cosine similarity of every module pair. The process of building the vector included the following 3 steps: 1) identifying the disease caused by one protein within the module, 2) searching the phenotypes induced by the disease obtained in step 1, and 3) constructing the vector, initializing the values with zero and subsequently updating the value of the phenotype vector according to the phenotype. The phenotype vectors *V*_*A*_ and *V*_*B*_ obtained for modules A and B were created, respectively, and the cosine of Equation 6 was used to calculate the similarity. The hypothesis that a shorter distance was associated with the most similar phenotype between two modules was tested by initially constructing the phenotype vector for each module as follows: 1) identifying the disease-related proteins located in one common module; 2) searching for all phenotypes induced by one disease; and 3) building the phenotype vector with elements equal to the number of phenotypes. The vector creation process is presented in Supplementary Fig. 3. Next, we used the formula in Equation 5, which was inspired by a previously published study^8^, to calculate the distance of two modules, followed by Equation (6), which was used to obtain the biological similarity between the two phenotype vectors that corresponded to the two modules.

6 $$cos({V}_{A},{V}_{B})=\frac{{V}_{A}\,\ast \,{V}_{B}}{\sqrt{|{V}_{A}|}\sqrt{|{V}_{B}|}}$$

## Electronic supplementary material

Supplementary Figures and Tables
