# Supplementary material for: Functional diversity of topological modules in human protein-protein interaction networks
Source: Sci Rep. 2017 Nov 23;7:16199. doi: 10.1038/s41598-017-16270-z (PMC5701033; doi:10.1038/s41598-017-16270-z)
Supplement: Supplementary file 1 — Supplementary Figures and Tables [file 41598_2017_16270_MOESM1_ESM.pdf]

Supplementary Figures and Tables

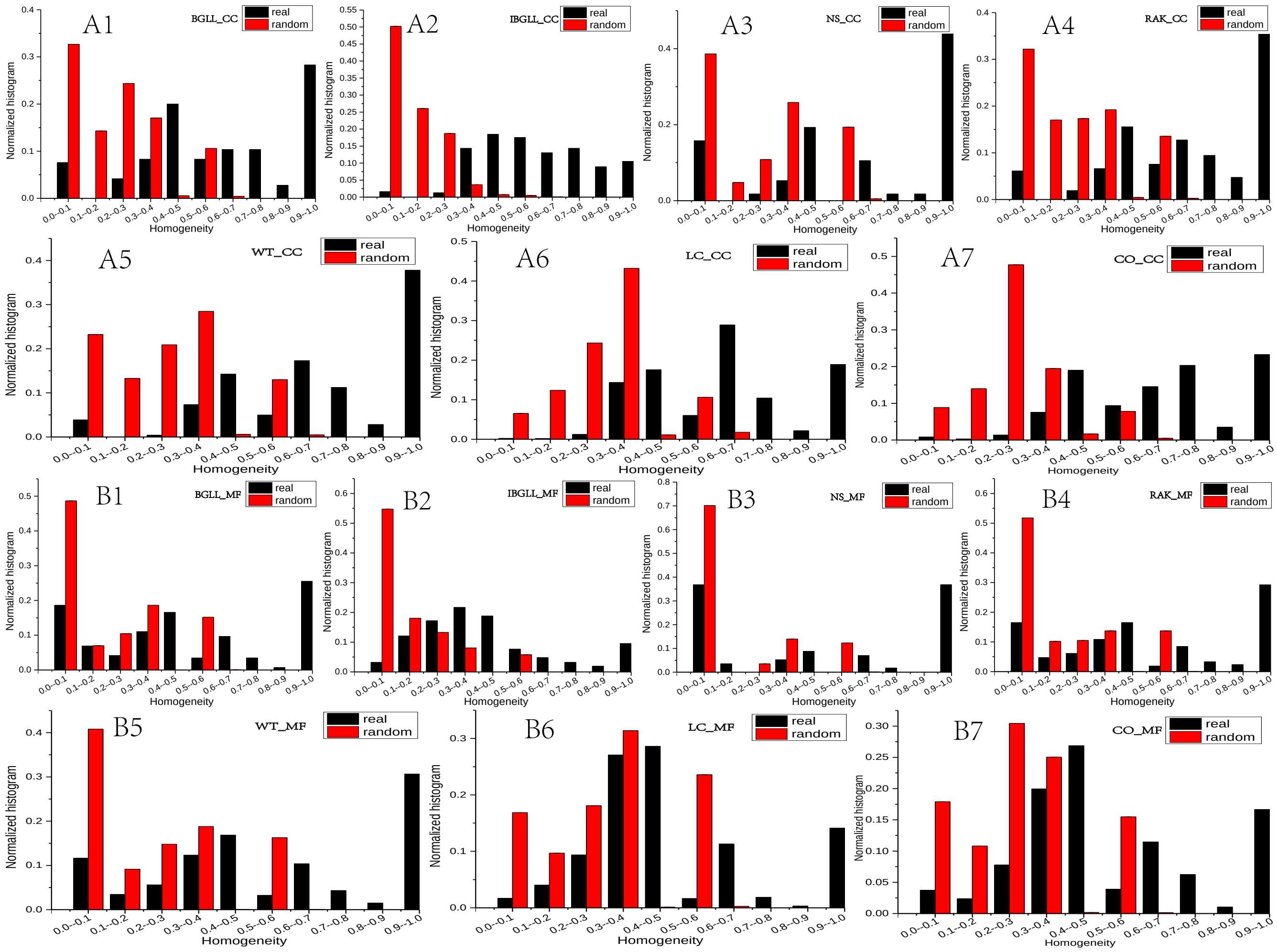

Figure 1. **Homogeneity of CC and MF associations at different module sizes.**

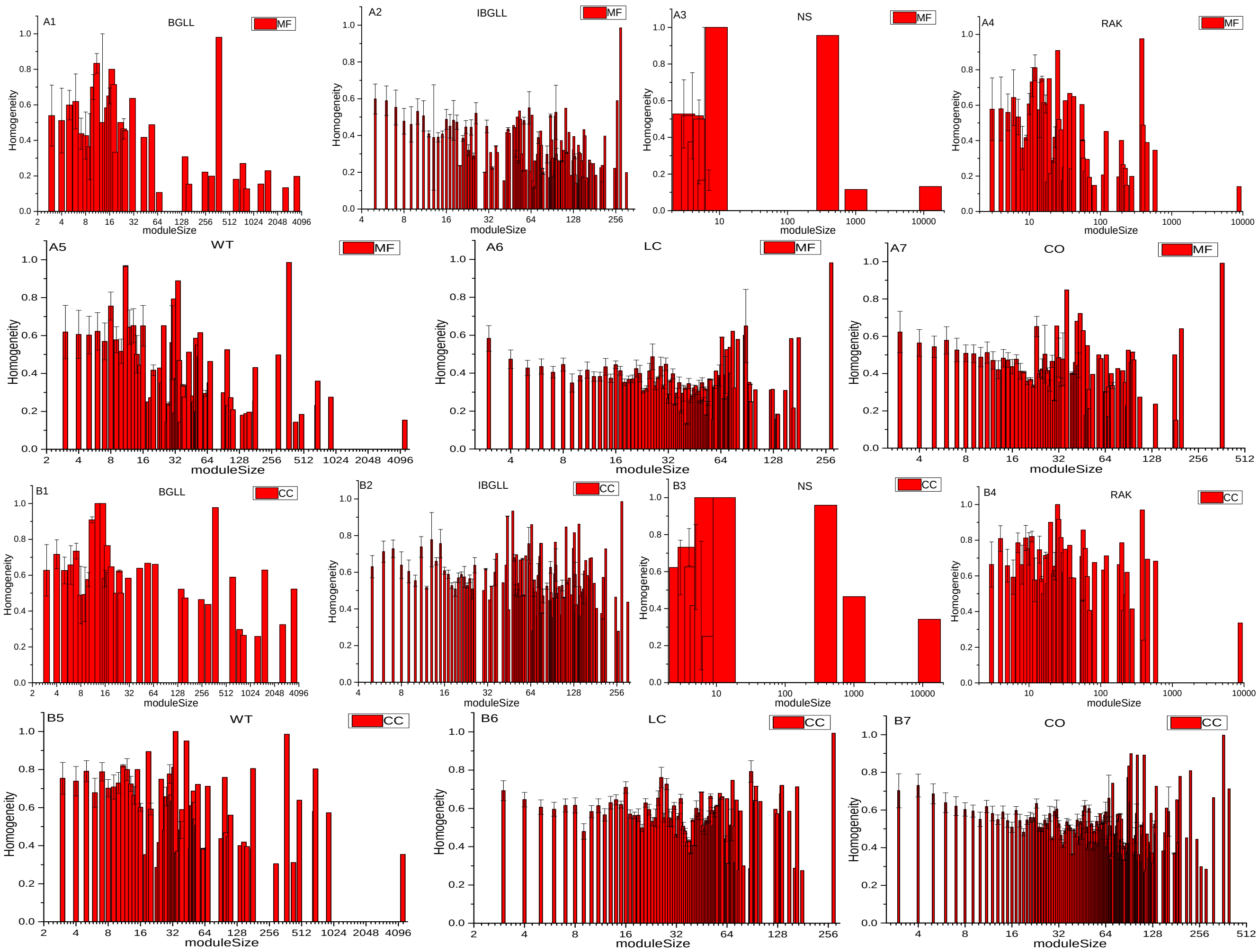

Figure 2. Homogeneity of MF and CC associations at different module sizes.

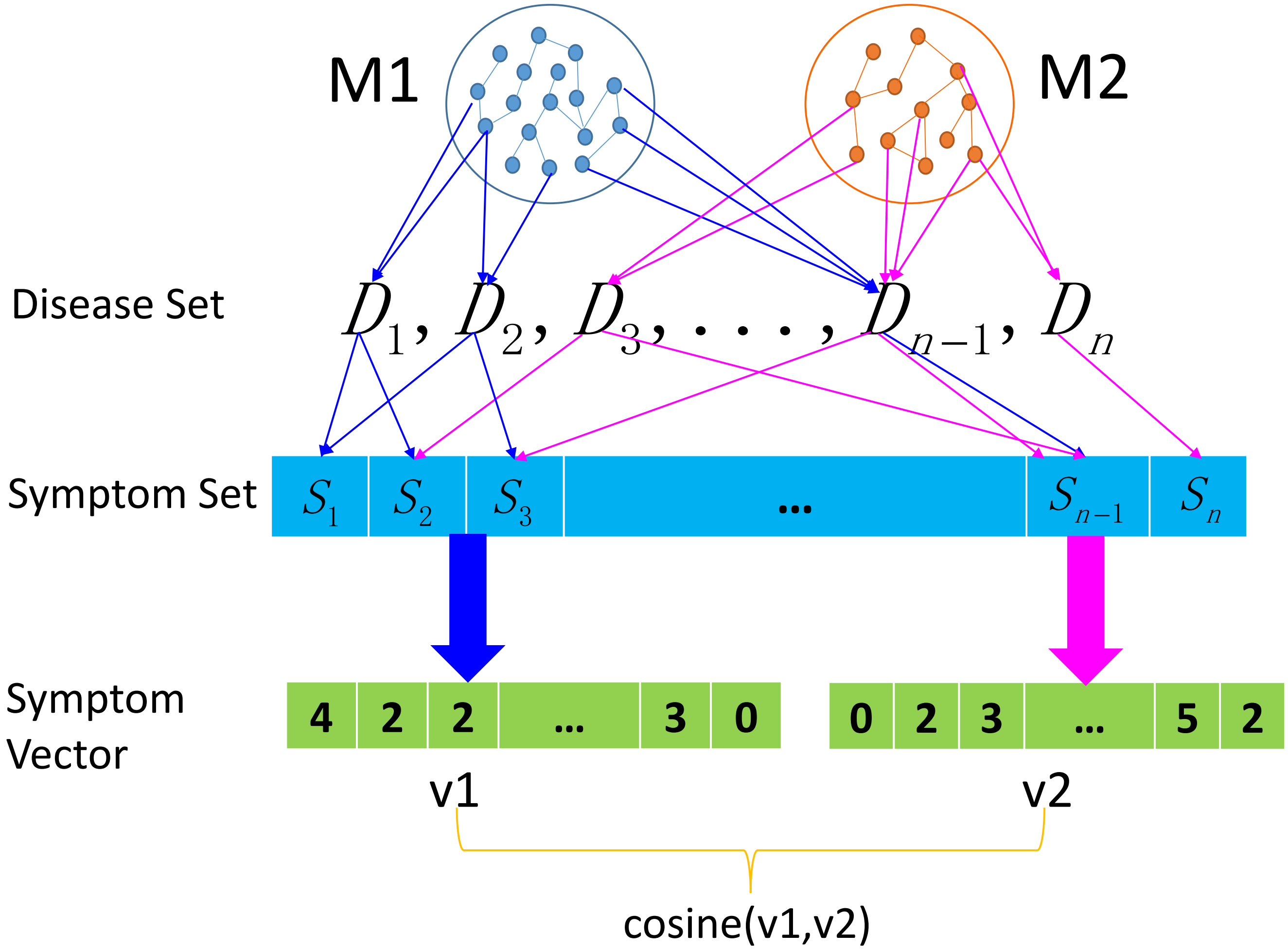

Figure 3. **The process of symptom vector constructed for a module.**

Method	BP	CC	MF	Pathway
BGLL	−0.21(0.01)	−0.16(0.05)	−0.17(0.04)	0.16(0.04)
RAK	−0.14(0.05)	−0.07(0.31)	−0.11(0.10)	0.26(1.40E−04)
NS	0.10(0.45)	0.11(0.41)	0.13(0.32)	0.48(1.98E−03)
WT	−0.15(0.01)	−0.09(0.03)	−0.13(0.09)	0.07(1.33E−03)

Table 1. Correlation between module size and homogeneity. PCC(p-value)

Method	BP	CC	MF	Pathway
BGLL	0.15(0.07)	0.16(0.04)	0.11(0.21)	-0.31(1.51E-04)
RAK	0.11(0.12)	0.15(0.03)	0.16(0.02)	-0.15(2.27E-03)
NS	-0.17(0.21)	0.07(0.61)	0.09(0.50)	-0.16(2.34E-04)
WT	0.18(6.93E-05)	0.19(2.76E-05)	0.11(0.02)	-0.23(6.59E-07)

Table 2. Correlation between edge density and homogeneity. PCC(p-value)

Method	PCC(p-value)
BGLL	0.44(<1E-127)
RAK	0.39(<1E-127)
NS	0.45(4.49E-76)
WT	0.31(<1E-127)

Table 3. Correlation between module distance and phenotypic similarity.PCC(p-value)

Method	BP	CC	MF	Pathway
BGLL	0.06(0.45)	0.06(0.46)	0.01(0.93)	0.04(0.58)
RAK	0.04(0.48)	0.05(0.45)	8.18E-03(0.91)	0.11(0.65)
NS	0.12(0.35)	0.08(0.56)	4.75E-03(0.76)	0.02(0.86)
WT	0.09(0.04)	0.07(0.12)	0.07(0.19)	0.09(0.05)

Table 4. Correlation between disease ratio and homogeneity. PCC(p-value)

number of proteins annotated by (or participated in) r GO terms (pathways)		PCC(p-value)			
		BP	CC	MF	pathway
BGLL	1	0.40(7.55E-07)	0.31(2.04E-04)	0.30(3.14E-04)	0.78(1.28E-29)
	2	0.25(2.29E-03)	0.13(0.12)	0.36(8.63E-06)	0.36(1.07E-05)
	3	0.14(0.09)	0.02(0.84)	0.22(8.41E-03)	0.25(0.00326)
	4	0.10(0.23)	0.11(0.20)	-0.01(0.95)	0.06(0.47)
	>=5	-0.049(0.56)	0.02(0.86)	-0.02(0.78)	0.28(7.75E-04)
RAK	1	0.42(2.44E-10)	0.15(0.03)	0.30(7.91E-06)	0.81(2.93E-50)
	2	0.24(3.64E-4)	0.15(0.03)	0.22(1.23E-03)	0.29(1.44E-05)
	3	0.14(0.04)	0.07(0.30)	0.21(1.92E-03)	0.22(1.13E-03)
	4	0.14(0.04)	0.11(0.09)	0.09(0.17)	0.26(1.43E-04)
	>=5	0.01(0.88)	0.10(0.14)	0.07(0.32)	0.25(2.84E-04)
NS	1	0.59(1.62E-06)	0.37(6.0E-03)	0.56(9.51E-06)	0.90(7.38E-21)
	2	0.31(0.02)	0.27(0.04)	0.41(2.14E-03)	NaN
	3	0.19(0.16)	0.06(0.67)	0.27(0.05)	NaN
	4	0.12(0.40)	0.19(0.16)	-2.16E-03(0.98)	0.41(1.88E-03)
	>=5	0.14(0.30)	9.72E-02(0.48)	0.05(0.71)	0.41(1.88E-03)
WT	1	0.27(5.72E-09)	0.11(0.02)	0.31(9.31E-12)	0.81(5.2E-17))
	2	0.18(7.68E-05)	0.10(0.03)	0.18(1.28E-04)	0.37(1.99E-16)
	3	0.05(0.29)	0.07(0.11)	0.09(0.05)	0.21(6.03E-06)
	4	0.089(0.06)	0.09(0.06)	0.07(0.13)	0.16(6.51E-04)
	>=5	7.83E-03(0.87)	0.04(0.35)	0.02(0.71)	0.14(3.10E-03)

Table 5. Correlation between the number of GO terms at different levels and homogeneity in terms of BP, CC, MF and pathway. PCC(p-value)
